# Quantitative Analysis of Retinal Perfusion in Patients with Frontotemporal Dementia Using Optical Coherence Tomography Angiography

**DOI:** 10.3390/diagnostics14020211

**Published:** 2024-01-18

**Authors:** Eliane Luisa Esser, Larissa Lahme, Sebastian Dierse, Raphael Diener, Nicole Eter, Heinz Wiendl, Thomas Duning, Matthias Pawlowski, Julia Krämer, Maged Alnawaiseh

**Affiliations:** 1 Department of Ophthalmology, University Hospital Münster, Albert- Schweitzer-Campus 1, Building D15, 48149 Münster, Germanymaged.alnawaiseh@klinikumbielefeld.de (M.A.); 2 Department of Neurology with Institute of Translational Neurology, University Hospital Münster, Albert-Schweitzer-Campus 1, Building A1, 48149 Münster, Germany; 3 Department of Neurology, Klinikum Bremen-Ost, 28325 Bremen, Germany; 4 Department of Ophthalmology, Klinikum Bielefeld, 33604 Bielefeld, Germany

**Keywords:** frontotemporal dementia, flow density, optical coherence tomography angiography, retinal and optic nerve head perfusion

## Abstract

Background: Optical coherence tomography angiography (OCT-A) provides detailed visualization of the perfusion of the vascular network of the eye. While in other forms of dementia, such as Alzheimer’s disease and mild cognitive impairment, reduced retinal perfusion was frequently reported, data of patients with frontotemporal dementia (FTD) are lacking. Objective: Retinal and optic nerve head perfusion was evaluated in patients with FTD with OCT-A. Quantitative OCT-A metrics were analyzed and correlated with clinical markers and vascular cerebral lesions in FTD patients. Methods: OCT-A was performed in 18 eyes of 18 patients with FTD and 18 eyes of 18 healthy participants using RTVue XR Avanti with AngioVue. In addition, patients underwent a detailed ophthalmological, neurological, and neuropsychological examination, cerebral magnetic resonance imaging (MRI), and lumbar puncture. Results: The flow density in the optic nerve head (ONH) and in the superficial capillary plexus (SCP) of the macula of patients was significantly lower compared to that of healthy controls (*p* < 0.001). Similarly, the VD in the deep capillary plexus (DCP) of the macula of patients was significantly lower compared to that of healthy controls (*p* < 0.001). There was no significant correlation between the flow density data, white matter lesions in brain MRI, cognitive deficits, and cerebrospinal fluid markers of dementia. Conclusions: Patients with FTD showed a reduced flow density in the ONH, and in the superficial and deep retinal capillary plexus of the macula, when compared with that of healthy controls. Quantitative analyses of retinal perfusion using OCT-A may therefore help in the diagnosis and monitoring of FTD. Larger and longitudinal studies are necessary to evaluate if OCT-A is a suitable biomarker for patients with FTD.

## 1. Introduction

Frontotemporal dementia (FTD) stands as a formidable challenge in the landscape of clinical diagnosis, marked by its intricate clinical presentations and a propensity for overlapping features with other forms of dementia [1,2]. The three recognized variants, encompassing the behavioral variant (bvFTD) and the semantic and non-fluent variants of primary progressive aphasia (PPP), contribute significantly to the clinical heterogeneity witnessed in individuals affected by FTD [1]. This complexity is further compounded by the potential co-occurrence of FTD with amyotrophic lateral sclerosis (ALS), introducing an additional layer of intricacy to the diagnostic milieu [2].

Despite ranking as the third most prevalent form of dementia across all age groups, trailing behind Alzheimer’s disease and Lewy body dementia, FTD garners particular attention for its prevalence as the second most common cause of early-onset dementia, surpassed only by Alzheimer’s disease. The early onset of FTD and its distinct symptomatology emphasize the urgent need for precise diagnostic tools, especially given the unique challenges associated with the manifestation of this condition in relatively younger individuals [3,4].

Nevertheless, achieving an accurate diagnosis remains a formidable challenge in the realm of FTD. The absence of specific and easily accessible biomarkers, coupled with the often invasive and expensive nature of existing diagnostic approaches, underscores the necessity for innovative and more accessible diagnostic methods. Recent studies have thus turned their focus toward exploring the potential of retinal changes as a non-invasive and cost-effective avenue for identifying FTD [5].

Researchers have discerned degenerative retinal changes in individuals with FTD, specifically noting a discernible thinning of the outer retinal layers when compared to those of healthy controls [6,7]. This thinning, notably occurring in the outer retina, emerges as a potential biomarker for FTD, imparting a distinctive feature that sets it apart from other forms of dementia [6,7]. For instance, Alzheimer’s disease is conventionally associated with thinning in the inner retina, highlighting the specificity of retinal changes in FTD [8,9,10,11].

In a proactive effort to advance our understanding of FTD and enhance diagnostic capabilities, this study strategically employs optical coherence tomography angiography (OCT-A). This cutting-edge technology for retinal imaging offers not only high-resolution images of retinal vessels but also a non-invasive, reproducible, and swift imaging technique [12,13,14,15]. Beyond visualization, OCT-A facilitates a quantitative analysis of blood flow in both the retina and the optic nerve head (ONH), presenting a comprehensive approach to unraveling the intricacies of FTD.

The primary objective of this study is to meticulously evaluate retinal and ONH perfusion in individuals diagnosed with FTD through the utilization of OCT-A. By harnessing the capabilities of this advanced imaging technology, researchers aspire not only to shed light on the potential of OCT-A measurements as quantitative indicators in FTD diagnosis but also to establish correlations between these measurements and the clinical or paraclinical markers associated with FTD.

In conclusion, this study represents a significant leap forward in the exploration of innovative and non-invasive diagnostic pathways for FTD. The integration of OCT-A holds promise not only in providing valuable insights into retinal changes associated with FTD but also in offering a novel approach to enhance diagnostic accuracy. Furthermore, this study is poised to contribute significantly to our evolving understanding of the pathophysiology underlying FTD, bridging critical gaps in our knowledge of this complex neurodegenerative disorder.

## 2. Materials and Methods

### 2.1. Participants

In total, this study comprised 18 age-matched healthy controls and 18 FTD patients with cognitive deficits (Montreal Cognitive Assessment score (MoCA) ≥ 6 and ≤25 points). The most recent (2011) revision of the clinical diagnostic criteria was used to diagnose FTD [16,17]. 

Patients were gathered from the University Hospital Muenster’s Department of Neurology in Germany. The University of Muenster’s multidisciplinary ethics committee and the Westphalian Physicians’ Chamber (Aerztekammer Westfalen-Lippe, Number 2015-402-f-S, 07.09.2015) gave their approval for the study. Both written and spoken information on the study’s contents was given to the patients. Prior to taking part in the trial, each individual provided written informed consent.

Excluded from the study were patients with neurological conditions other than frontotemporal dementia (FTD), including other forms of dementia, neurodegenerative disorders (e.g., motor neuron disease, multiple system atrophy, and Huntington’s disease), stroke, hydrocephalus, epilepsy, head injury, malignant, infectious, or inflammatory diseases, psychiatric illnesses unrelated to the dementia process (e.g., drug or alcohol abuse), and other systemic diseases that impair cognitive functioning. Patients with diabetic retinopathy or diabetes were excluded. Not a single patient had signs of an acute inflammatory process, such as increased neutrophil count, C-reactive protein, or erythrocyte sedimentation rate. Individuals who had undergone prior retinal surgery, had glaucoma or pre-existing macular diseases (e.g., age-related macular degeneration, macular hole or macular edema), or were potentially at risk for optic disc atrophy were not included in the study. Individuals who had thick cataracts or ocular abnormalities that prevented clear imaging were also disqualified.

### 2.2. Examinations

A skilled study physician (JK, MP, and TD) examined each patient physically and neurologically. In addition, each patient underwent a thorough neuropsychological evaluation, a family and medical history assessment, a lumbar puncture, an electroencephalogram, event-related potentials (P300), and magnetic resonance imaging (MRI) of the brain. Structural MRI analysis was carried out by two knowledgeable assessors who were blinded to the clinical data (JK and TD). MRIs were examined visually for lacunar infarction, moderate-to-severe white matter hyperintensities, and atrophy patterns suggestive of neurodegenerative disorders other than FTD, which were excluded. Vascular cerebral white matter lesions were scored using the widely accepted Fazekas et al. periventricular score on a 3-point rating system [18]. 

Along with refraction, best-corrected visual acuity, intraocular pressure measurement, anterior segment examination, and fundus examination, a comprehensive ophthalmic examination was also carried out. After that, each patient underwent OCT angiography (OCT-A) imaging of the optic nerve head (ONH) and macula, in the same place and under the same settings, which was performed by an expert examiner (ELE).

### 2.3. OCT Angiography

All individuals underwent OCTA imaging using RTVue XR Avanti with AngioVue (Optovue Inc., Fremont, CA, USA). With a light source centered at 840 nm and a bandwidth of 45 nm, this spectral-domain OCT system can perform 70,000 scans per second in the A-scan mode. The split-spectrum amplitude-decorrelation angiography algorithm was used to create OCT-A data. A detailed description of OCTA technology has been published elsewhere [19,20]. The essential principle is as follows, to put it briefly.

OCT B-scans obtained progressively at the same location will be identical in every way since the retina is a stationary object, with the exception of the blood’s movement within the retinal vessels. Hence, blood flow can be seen by comparing multiple OCTB images of the same retinal region and searching for pixel-by-pixel variations between the scans [20]. A 3 × 3 mm^2^ scan was used for OCTA imaging of the macula, and a 4.5 × 4.5 mm^2^ scan was used to acquire pictures of the peripapillary region and the optic nerve head (ONH). The study only included high-quality OCT-A images; photos including lines or gaps because of weak signal strength or motion artifacts were not included. Before data processing, the automated segmentation was verified.

### 2.4. MoCa Score

The Montreal Cognitive Assessment (MoCA) is a widely used cognitive screening tool designed to detect mild cognitive impairment (MCI) and early stages of dementia. It was developed by Dr. Ziad Nasreddine and his colleagues in 2005 as a brief but effective measure of cognitive function. The MoCA assesses a range of cognitive domains, providing a more comprehensive evaluation compared to some other cognitive screening instruments [21].

The assessment consists of various tasks that evaluate different cognitive abilities, including the following:

1. Visuospatial and Executive Functions: Assessing the ability to perceive spatial relationships and plan and execute tasks;
2. Naming: Evaluating language and semantic memory by asking individuals to name certain objects;
3. Memory: Testing short-term memory recall and recognition;
4. Attention: Assessing sustained attention and concentration;
5. Language: Evaluating language skills, including sentence repetition and verbal fluency;
6. Abstraction: Testing the ability to understand and interpret proverbs or similar conceptual tasks;
7. Delayed Recall: Measuring the ability to remember information after a short delay;
8. Orientation: Assessing awareness of person, place, and time.

The total score on the MoCA is 30 points, with a score of 26 or above generally considered normal. A lower score may indicate the presence of cognitive impairment [21].

### 2.5. Fazekas Score

The Fazekas scale is a scoring system used to quantify the extent of white matter lesions in the brain, particularly in the periventricular and subcortical regions. It is commonly employed to assess the severity of white matter changes on neuroimaging studies, providing valuable information in the context of cerebrovascular diseases and neurodegenerative disorders [18,22,23].

The scale categorizes white matter lesions on a scale from 0 to 3, with the following interpretations:

- 0: Absence of lesions;
- 1: Focal lesions;
- 2: Beginning of confluence of lesions;
- 3: Large confluent lesions

The Fazekas scale is often utilized in studies investigating cerebrovascular contributions to cognitive impairment and various neurological conditions.

### 2.6. Statistics

Microsoft Excel 2016 was used for data management. For statistical studies, GraphPad Prism 10.1 (GraphPad Software Inc., San Diego, CA, USA) and IBM SPSS^®^ Statistics 29 for Windows (IBM Corporation, Somers, NY, USA) were utilized. The Shapiro–Wilk test was used to determine whether or not the data were normally distributed, and the results showed that they were not. As a result, the two groups were compared using the Mann–Whitney U test for non-normally distributed variables, and Spearman’s correlation coefficient (rSp.) was used to express the degree of connection between two variables. The data are shown as median [25, 75 percentile] and mean ± SD. Interpretations of inferential statistics are meant to be exploratory (producing hypotheses), not confirmatory. A threshold of statistical significance of *p* < 0.05 was selected.

## 3. Results

In this prospective study, in total, eighteen patients diagnosed with frontotemporal dementia (FTD) and an equal number of age-matched control subjects were included. The patient group exhibited at least mild cognitive impairment, with a mean Montreal Cognitive Assessment (MoCA) score of 16.82 ± 6.70 (17.0 [11.0, 22.5]). The average white matter lesion load, as determined by MRI, was characterized as mild to moderate, with a mean Fazekas score of 0.95 ± 0.73 (1.0 [0.0, 1.25]). A comprehensive overview of the demographic and clinical characteristics of the participants is presented in Table 1.

Notably, there were no significant differences between the patient and control groups in terms of age (*p* = 0.56) or gender. Cerebrospinal fluid (CSF) data were available for all FTD patients, with the exception of two individuals who did not undergo lumbar puncture for diagnostic confirmation. Importantly, none of the patients exhibited CSF biomarker profiles indicative of Alzheimer’s disease, as evidenced by mean amyloid-β levels of 922.31 pg/mL ± 290.78 pg/mL and mean tau protein levels of 329.44 pg/mL ± 214.01 pg/mL.

Further characterization of the FTD patient subgroup revealed that five individuals were classified as behavioral variant FTD (bvFTD), four as having the semantic variant of primary progressive aphasia (PPA), and two as having the non-fluent variant of PPA. Additionally, two patients presented with FTD-ALS, while five exhibited an overlap between bvFTD and progressive supranuclear palsy (PSP).

### 3.1. OCT-A Findings

The analysis of flow density in the superficial capillary plexus (SCP) of the macula revealed consistent and statistically significant reductions across various regions compared to the control group. Noteworthy decreases were observed in the overall en face area (patients: 44.42 ± 2.82 (44.55 [43.03, 46.50]%); control group: 49.97 ± 3.72 (50.37 [45.77, 53.11]%); *p* < 0.001), the foveal area (patients: 19.09 ± 4.78 (18.85 [15.96, 22.38]%); control group: 28.42 ± 5.94 (29.82 [25.84, 33.15]%); *p* < 0.001), and the parafoveal area (patients: 47.10 ± 3.26 (47.85 [46.15, 49.38]%); control group: 52.07 ± 3.46 (52.42 [48.92, 55.08]%); *p* < 0.001).

Similarly, the flow density in the deep capillary plexus (SCP) of the macula exhibited significant reductions in patients with frontotemporal dementia (FTD) compared to that in the control group. This reduction was particularly evident in the deep foveal area (patients: 47.93 ± 3.64 (47.20 [45.80, 49.95]%); control group: 56.24 ± 2.56 (56.55 [54.75, 58.24]%); *p* < 0.001) and the parafoveal area (patients: 49.84 ± 3.86 (49.25 [48.35, 50.80]%); control group: 58.97 ± 2.95 (59.14 [57.53, 61.29]%); *p* < 0.001). Although the data for the foveal area approached significance (patients: 33.41 ± 6.44 (33.85 [27.20, 38.83]%); control group: 27.71 ± 6.28 (27.14 [24.25, 30.42]%); *p* = 0.006), the consistent trend of reduced flow density persisted.

**Figure 1:**
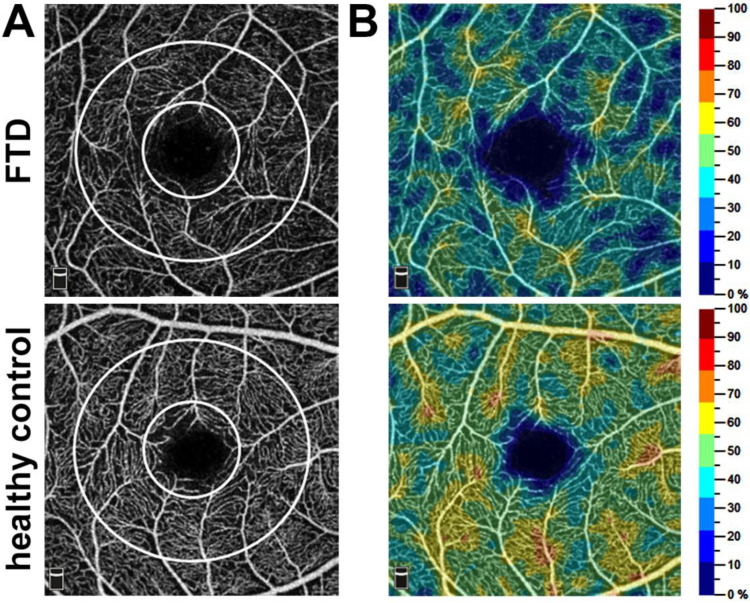
The macula (**A**) and color-coded vascular density maps (**B**) from superficial retinal OCT angiograms in a frontotemporal dementia (FTD) patient and a healthy control. Three different flow densities were analyzed: the parafoveal flow density (diameter from 1 mm to 2.5 mm), the whole en face flow density (average flow density of the complete 2.5 mm circle), and the foveal flow density (small white circle with a diameter of 1 mm).

**Figure 2:**
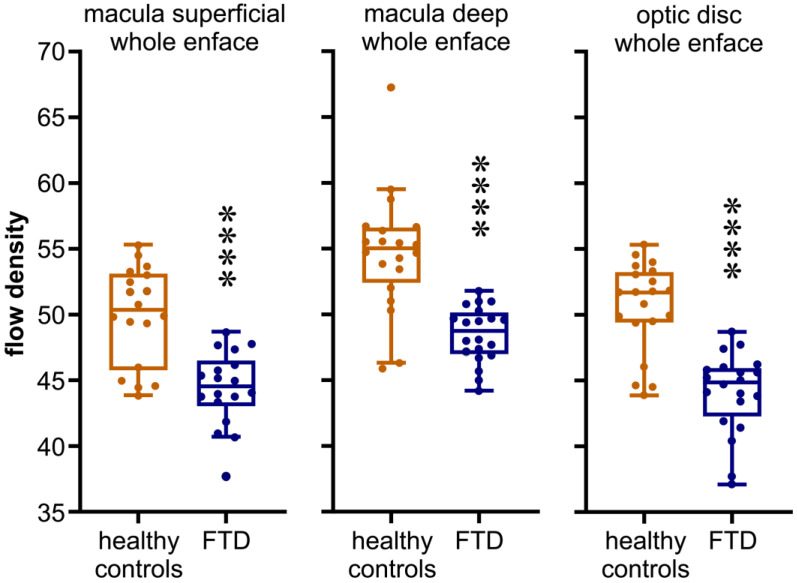
Comparison of the flow density (%) obtained in the superficial and deep capillary plexus of the macula and optic disc OCT angiogram in patients with FTD and healthy controls (*p* < 0.001). **** = statistically highly significant.

Additionally, patients with FTD exhibited a substantial reduction in flow density in the optic nerve head (ONH) compared to that of control subjects. This reduction was evident both in the overall en face region (patients: 48.08 ± 1.85 (47.95 [46.68, 49.55]%); control group: 58.97 ± 2.95 (59.14 [57.53, 61.29]%); *p* < 0.001) and the peripapillary region (patients: 50.69 ± 2.47 (50.5 [49.30, 52.53]%); control group: 62.17 ± 5.48 (63.53 [58.63, 65.71]%); *p* < 0.001) as illustrated in Figure 1 and Figure 2.

Flow density data of the optic nerve head and the superficial and deep capillary plexus of the macula are summarized in Table 2.

### 3.2. Association of OCT-A Alterations with Clinical and Paraclinical Findings

There were no significant correlations between the OCT-A metrics and the different CSF markers of dementia (amyloid-ß or tau levels). Similarly, no statistically significant associations were found between the OCT-A metrics and the MoCA score or the Fazekas score (Table 3).

## 4. Discussion

To the best of our knowledge, we are the first to investigate retinal perfusion using OCTA in patients with FTD, demonstrating a significantly reduced blood flow.

Our findings contribute to the evolving understanding of cerebrovascular involvement in FTD, extending beyond cognitive impairment to encompass broader neurovascular changes [24,25,26]. Utilizing advanced imaging techniques like single-photon emission computed tomography and arterial spin-labeling, previous studies have consistently demonstrated altered cerebral blood flow in FTD [24,27,28].

Notably, the study by Mutsearts and colleagues adds a compelling dimension by demonstrating deviations in cerebral blood flow even in presymptomatic stages of genetic frontotemporal dementia, shedding light on the potential involvement of vascular factors in the early phases of the disease [26].

The study revealed significant changes in cerebral perfusion in individuals in the presymptomatic stage of genetic frontotemporal dementia (FTD). These findings provide crucial insights into the early stages of the disease and contribute to a deeper understanding of the underlying pathophysiology. The work underscores the importance of imaging techniques for the early detection and understanding of neurodegenerative diseases, particularly genetically mediated FTD [26].

The convergence of pathophysiological, embryonic, and structural similarities between the brain and the eye propels the consideration of retinal alterations as effective and distinctive biomarkers for dementia [5,29]. 

The study by Bambo et al. investigated the correlations between visual function, retinal nerve fiber layer (RNFL) degeneration, and the severity of dementia in patients with Alzheimer’s disease. The researchers identified correlations between the severity of dementia and specific aspects of visual function and RNFL degeneration. The findings suggest that changes in retinal structure and function may be associated with the progression of Alzheimer’s disease [29].

### Formularbeginn

Optical coherence tomography angiography (OCT-A) has emerged as a revolutionary tool in this pursuit, enabling the quantitative analysis of retinal blood flow. The virtues of accuracy, reproducibility, and ease of application render OCT-A not only a valuable asset in ocular and systemic disease research but also a key contributor to the understanding of microcirculation dynamics, even extending its impact to various animal models [30,31,32,33].

Our study stands as a pioneer in the realm of FTD research by presenting a comprehensive analysis of decreased vascular perfusion in affected individuals. The examination spans both the superficial and deep capillary plexus of the macula, along with the optic nerve region, providing a nuanced understanding of microvascular changes. The noteworthy extension of reduced perfusion beyond the macula reinforces the hypothesis that diffuse microvascular alterations may serve as pivotal contributors to the development of FTD [28]. In doing so, the study introduces a new perspective on the intricate web of factors influencing FTD pathogenesis, elevating vascular alterations to a potential key player in the complex interplay leading to the manifestation of the disease.

The Fazekas scale, a widely recognized scoring system, assesses white matter lesions in both periventricular and subcortical regions on a scale ranging from 0 to 3. This scale plays a pivotal role in diagnosing vascular components during the diagnostic workup, providing valuable insights into the extent and nature of white matter lesions. Its application significantly influences the diagnostic process, aiding in the identification and understanding of potential vascular factors as part of the overall clinical evaluation [18,22,23].

In the course of our study, we did not discern a notable correlation between the decline in flow density and the augmentation of white matter lesions. This observed incongruity might be ascribed to the predominant presence of patients exhibiting a Fazekas score of 1.

Furthermore, the absence of a significant correlation between retinal perfusion metrics and the Fazekas score raises intriguing questions about the specific vascular components contributing to retinal alterations in FTD. The multifaceted nature of FTD, which involves a complex interplay of vascular, neurodegenerative, and inflammatory factors, complicates the straightforward correlation between retinal perfusion and traditional markers of dementia.

The absence of significant correlations between the OCT-A metrics and various cerebrospinal fluid (CSF) markers of dementia, including levels of amyloid-ß or tau, underscores the complexity of the relationship between retinal perfusion and biochemical markers in the context of frontotemporal dementia (FTD). While OCT-A has demonstrated its potential as a valuable tool for assessing retinal microcirculation, its lack of direct correlation with specific CSF markers suggests that the underlying pathophysiological processes in the retina may not directly mirror those in the cerebrospinal fluid.

Certain investigations have established a correlation between retinal thickness and performance on neuropsychological assessments, suggesting a potential direct association between retinal structure as measured by OCT and cognitive function [34]. The Montreal Cognitive Assessment (MoCA), serving as a cognitive deficit screening tool, encompasses a spectrum of cognitive abilities, including language, abstraction, memory, executive functions, visuospatial construction, and attention [35,36]. Scores on the MoCA can range from 0 to 30 [35,36]. However, the absence of a discernible correlation between vascular changes and cognitive deficits in our cohort may be attributed to the considerable clinical heterogeneity among our study participants. Lastly, it is noteworthy that diminished ocular blood flow could contribute to retinal thinning, as evidenced in several studies within the FTD literature [3,6,7].

In Kim et al.’s study, optical coherence tomography (OCT) identified outer retina thinning in patients with frontotemporal degeneration (FTD). These findings suggest that OCT may capture changes in retinal structure as a potential marker for FTD [7].

Our study is subject to certain limitations. Firstly, the sample size is relatively small, which could impact the generalizability of our findings. Secondly, the study design is cross-sectional, limiting our ability to draw conclusions about the utility of flux density measurements in gauging disease progression over time. To address these limitations and enhance the robustness of our findings, future research should prioritize longitudinal studies with larger and more diverse cohorts. These studies could include comparisons between various FTD subtypes and patients with vascular dementia.

In conclusion, our study contributes to the understanding of altered microcirculation in the macula and optic nerve among FTD patients. The quantitative analysis of retinal perfusion using OCT-A emerges as a promising, non-invasive, and objective diagnostic tool for FTD. This research not only advances our diagnostic capabilities but also underscores the potential of OCT-A in monitoring microcirculation and central perfusion in individuals grappling with neurodegenerative diseases. As we navigate the complex landscape of FTD research, these insights pave the way for the further exploration and refinement of diagnostic methodologies.

## Figures and Tables

**Table 1:** Demographics and clinical characteristics of participants.

	Patients	Healthy Controls	*p* Value
Subjects, n	18	18	
Gender (m/f), n	10/8	10/8	
Age (y)	65.67 ± 6.82 (66 [61.25, 70.25])	66.67 ± 7.87 (69 [58.50, 73.25])	0.56
Spherical equivalent (D)	0.84 ± 1.79 (0.56 [−0.38, 1.28])	0.76 ± 1.45 (0.44 [−0.16, 1.22])	0.83
IOP (mmHg)	16.11 ± 3.32 (16 [14.75,18.00])	15.61 ± 2.38 (15 [14.75, 17.25])	0.72
Visual acuity (LogMar notation)	0.06 ± 0.1 (0.0 [0.0, 0.1])	0.06 ± 0.1 (0.5 [0.0, 0.1])	0.88
CSF amyloid ß (pg/mL)	922.31 ± 290.78 (971.50 [671.00, 1142.75])		
CSF total tau protein (pg/mL)	329.44 ± 214.01 (273.50 [188.75, 393.75])		
Fazekas score	0.95 ± 0.73 (1.0 [0.0, 1.25])		
MoCA score	16.82 ± 6.70 (17.0 [11.0, 22.5])		

All values except the number of participants and the number of male and female participants are mean values with standard deviation (SD) and median values with 25, 75 percentiles. CSF = cerebrospinal fluid; D = diopter; IOP = intraocular pressure; n = number; m = male, f = female, y = years; MoCA = Montreal Cognitive Assessment.

**Table 2:** Flow density data of the superficial and deep capillary plexus of the macula and optic nerve head.

	Patients	Healthy Controls	*p* Value
OCT-A SCP			
whole en face	44.42 ± 2.82 (44.55 [43.03, 46.50])	49.97 ± 3.72 (50.37 [45.77, 53.11])	<0.001
fovea	19.09 ± 4.78 (18.85 [15.96, 22.38])	28.42 ± 5.94 (29.82 [25.84, 33.15])	<0.001
parafoveal	47.10 ± 3.26 (47.85 [46.15, 49.38])	52.07 ± 3.46 (52.42 [48.92, 55.08])	<0.001
OCT-A DCP			
whole en face	47.93 ± 3.64 (47.20 [45.80, 49.95])	56.24 ± 2.56 (56.55 [54.75, 58.24])	<0.001
fovea	33.41 ± 6.44(33.85 [27.20, 38.83])	27.71 ± 6.28(27.14 [24.25, 30.42])	0.006
parafoveal	49.84 ± 3.86 (49.25 [48.35, 50.80])	58.97 ± 2.95 (59.14 [57.53, 61.29])	<0.001
OCT-A ONH			
whole en face	48.08 ± 1.85 (47.95 [46.68, 49.55])	54.57 ± 4.90 (55.03 [50.92, 56.68])	<0.001
peripapillary	50.69 ± 2.47 (50.5 [49.30, 52.53])	62.17 ± 5.48 (63.53 [58.63, 65.71])	<0.001

All values are mean values with standard deviation (SD) and median values with 25, 75 percentiles; OCT-A = optical coherence tomography angiography; SCP = superficial capillary plexus; DCP = deep capillary plexus; ONH = optic nerve head.

**Table 3:** Correlations between the flow density in the superficial OCT angiogram of the macula and clinical and paraclinical findings.

		Flow Density Whole En Face	Flow Density Parafoveal
CSF amyloid-ß (pg/mL)	Spearman’s r	0.200	0.060
	*p*-Value	0.458	0.824
CSF total tau protein (pg/mL)	Spearman’s r	0.365	0.456
	*p*-Value	0.165	0.076
MoCA score	Spearman’s r	0.207	0.054
	*p*-Value	0.425	0.836
Fazekas score	Spearman’s r	−0.221	−0.161
	*p*-value	0.378	0.524

CSF = cerebrospinal fluid; MoCA = Montreal Cognitive Assessment.

## Data Availability

The raw data supporting the conclusions of this article will be made available by the authors on request.

## References

[1] Bang J., Spina S., Miller B.L. (2015). Frontotemporal dementia. Lancet.

[2] Boeve B.F., Boxer A.L., Kumfor F., Pijnenburg Y., Rohrer J.D. (2022). Advances and controversies in frontotemporal dementia: Diagnosis, biomarkers, and therapeutic considerations. Lancet Neurol..

[3] Ferrari L., Huang S.-C., Magnani G., Ambrosi A., Comi G., Leocani L. (2017). Optical Coherence Tomography Reveals Retinal Neuroaxonal Thinning in Frontotemporal Dementia as in Alzheimer’s Disease. J. Alzheimer’s Dis..

[4] Krämer J., Lueg G., Schiffler P., Vrachimis A., Weckesser M., Wenning C., Pawlowski M., Johnen A., Teuber A., Wersching H. (2018). Diagnostic Value of Diffusion Tensor Imaging and Positron Emission Tomography in Early Stages of Frontotemporal Dementia. J. Alzheimer’s Dis..

[5] Moinuddin O., Khandwala N.S., Young K.Z., Sathrasala S.K., Barmada S.J., Albin R.L., Besirli C.G. (2021). Role of Optical Coherence Tomography in Identifying Retinal Biomarkers in Frontotemporal Dementia: A Review. Neurol. Clin. Pract..

[6] Kim B.J., Grossman M., Song D., Saludades S., Pan W., Dominguez-Perez S., Dunaief J.L., Aleman T.S., Ying G.S., Irwin D.J. (2019). Persistent and Progressive Outer Retina Thinning in Frontotemporal Degeneration. Front. Neurosci..

[7] Kim B.J., Irwin D.J., Song D., Daniel E., Leveque J.D., Raquib A.R., Pan W., Ying G.S., Aleman T.S., Dunaief J.L. (2017). Optical coherence tomography identifies outer retina thinning in frontotemporal degeneration. Neurology.

[8] Chan V.T., Sun Z., Tang S., Chen L.J., Wong A., Tham C.C., Wong T.Y., Chen C., Ikram M.K., Whitson H.E. (2019). Spectral-Domain OCT Measurements in Alzheimer’s Disease: A Systematic Review and Meta-analysis. Ophthalmology.

[9] Cheung C.Y., Mok V., Foster P.J., Trucco E., Chen C., Wong T.Y. (2021). Retinal imaging in Alzheimer’s disease. J. Neurol. Neurosurg. Psychiatry.

[10] Cheung C.Y.L., Ong Y.T., Hilal S., Ikram M.K., Low S., Ong Y.L., Venketasubramanian N., Yap P., Seow D., Chen C.L.H. (2015). Retinal ganglion cell analysis using high-definition optical coherence tomography in patients with mild cognitive impairment and Alzheimer’s disease. J. Alzheimer’s Dis..

[11] Coppola G., Di Renzo A., Ziccardi L., Martelli F., Fadda A., Manni G., Barboni P., Pierelli F., Sadun A.A., Parisi V. (2015). Optical Coherence Tomography in Alzheimer’s Disease: A Meta-Analysis. PLoS ONE.

[12] López-Cuenca I., Salobrar-García E., Sánchez-Puebla L., Espejel E., García del Arco L., Rojas P., Elvira-Hurtado L., Fernández-Albarral J.A., Ramírez-Toraño F., Barabash A. (2022). Retinal Vascular Study Using OCTA in Subjects at High Genetic Risk of Developing Alzheimer’s Disease and Cardiovascular Risk Factors. J. Clin. Med..

[13] Hosari S., Hohberger B., Theelke L., Sari H., Lucio M., Mardin C.Y. (2020). OCT Angiography: Measurement of Retinal Macular Microvasculature with Spectralis II OCT Angiography—Reliability and Reproducibility. Ophthalmologica.

[14] Alnawaiseh M. (2019). Optische Kohärenztomographie-Angiographie zur Beurteilung der Mikrozirkulation bei systemischen Erkrankungen. Ophthalmologe.

[15] Lahme L., Esser E.L., Mihailovic N., Schubert F., Lauermann J., Johnen A., Eter N., Duning T., Alnawaiseh M. (2018). Evaluation of Ocular Perfusion in Alzheimer’s Disease Using Optical Coherence Tomography Angiography. J. Alzheimer’s Dis..

[16] Gorno-Tempini M.L., Hillis A.E., Weintraub S., Kertesz A., Mendez M., Cappa S.F., Ogar J.M., Rohrer J.D., Black S., Boeve B.F. (2011). Classification of primary progressive aphasia and its variants. Neurology.

[17] Rascovsky K., Hodges J.R., Knopman D., Mendez M.F., Kramer J.H., Neuhaus J., Van Swieten J.C., Seelaar H., Dopper E.G., Onyike C.U. (2011). Sensitivity of revised diagnostic criteria for the behavioural variant of frontotemporal dementia. Brain.

[18] Scheltens P., Erkinjunti T., Leys D., Wahlund L.O., Inzitari D., del Ser T., Pasquier F., Barkhof F., Mäntylä R., Bowler J. (1998). White matter changes on CT and MRI: An overview of visual rating scales. European Task Force on Age-Related White Matter Changes. Eur. Neurol..

[19] Foulsham W., Chien J., Lenis T.L., Papakostas T.D. (2022). Optical Coherence Tomography Angiography: Clinical Utility and Future Directions. J. VitreoRetin. Dis..

[20] Spaide R.F., Fujimoto J.G., Waheed N.K., Sadda S.R., Staurenghi G. (2018). Optical coherence tomography angiography. Prog. Retin. Eye Res..

[21] Nasreddine Z.S., Phillips N.A., Bédirian V., Charbonneau S., Whitehead V., Collin I., Cummings J.L., Chertkow H. (2005). The Montreal Cognitive Assessment, MoCA: A brief screening tool for mild cognitive impairment. J. Am. Geriatr. Soc..

[22] Fazekas F., Chawluk J.B., Alavi A., Hurtig H.I., Zimmerman R.A. (1987). MR signal abnormalities at 1.5 T in Alzheimer’s dementia and normal aging. AJR Am. J. Roentgenol..

[23] Verhagen M.V., Guit G.L., Hafkamp G.J., Kalisvaart K. (2016). The impact of MRI combined with visual rating scales on the clinical diagnosis of dementia: A prospective study. Eur. Radiol..

[24] Tosun D., Rosen H., Miller B.L., Weiner M.W., Schuff N. (2012). MRI patterns of atrophy and hypoperfusion associations across brain regions in frontotemporal dementia. Neuroimage.

[25] Iadecola C. (2010). The overlap between neurodegenerative and vascular factors in the pathogenesis of dementia. Acta Neuropathol..

[26] Mutsaerts H.J., Mirza S.S., Petr J., Thomas D.L., Cash D.M., Bocchetta M., De Vita E., Metcalfe A.W., Shirzadi Z., Robertson A.D. (2019). Cerebral perfusion changes in presymptomatic genetic frontotemporal dementia: A GENFI study. Brain.

[27] Ssali T., Anazodo U.C., Narciso L., Liu L., Jesso S., Richardson L., Günther M., Konstandin S., Eickel K., Prato F. (2022). Sensitivity of Arterial Spin Labeling for Characterization of Longitudinal Perfusion Changes in Frontotemporal Dementia and Related Disorders. Neuroimage Clin..

[28] Du A.T., Jahng G.H., Hayasaka S., Kramer J.H., Rosen H.J., Gorno-Tempini M.L., Rankin K.P., Miller B.L., Weiner M.W., Schuff N. (2006). Hypoperfusion in frontotemporal dementia and Alzheimer disease by arterial spin labeling MRI. Neurology.

[29] Bambo M.P., Garcia-Martin E., Otin S., Pinilla J., Larrosa J.M., Polo V., Pablo L.E. (2015). Visual function and retinal nerve fibre layer degeneration in patients with Alzheimer disease: Correlations with severity of dementia. Acta Ophthalmol..

[30] Wang X.N., Zhao Q., Li D.J., Wang Z.Y., Chen W., Li Y.F., Cui R., Shen L., Wang R.K., Peng X.Y. (2019). Quantitative evaluation of primary retinitis pigmentosa patients using colour Doppler flow imaging and optical coherence tomography angiography. Acta Ophthalmol..

[31] Alnawaiseh M., Brand C., Bormann E., Wistuba J., Eter N., Heiduschka P. (2017). Quantitative analysis of retinal perfusion in mice using optical coherence tomography angiography. Exp. Eye Res..

[32] Brand C., Zitzmann M., Eter N., Kliesch S., Wistuba J., Alnawaiseh M., Heiduschka P. (2017). Aberrant ocular architecture and function in patients with Klinefelter syndrome. Sci. Rep..

[33] Lange P.S., Mihailovic N., Esser E., Frommeyer G., Fischer A.J., Bode N., Höwel D., Rosenberger F., Eter N., Eckardt L. (2021). Improvement of Retinal Microcirculation after Pulmonary Vein Isolation in Patients with Atrial Fibrillation—An Optical Coherence Tomography Angiography Study. Diagnostics.

[34] Oktem E.O., Derle E., Kibaroglu S., Oktem C., Akkoyun I., Can U. (2015). The relationship between the degree of cognitive impairment and retinal nerve fiber layer thickness. Neurol. Sci..

[35] Freitas S., Simões M.R., Alves L., Duro D., Santana I. (2012). Montreal Cognitive Assessment (MoCA): Validation study for frontotemporal dementia. J. Geriatr. Psychiatry Neurol..

[36] Deutsch M.B., Liang L.-J., Jimenez E.E., Mather M.J., Mendez M.F. (2016). Are we comparing frontotemporal dementia and Alzheimer disease patients with the right measures?. Int. Psychogeriatr..

